# Rediscovery of *Rhyacoglanis pulcher* (Boulenger, 1887) (Siluriformes: Pseudopimelodidae), a rare rheophilic bumblebee catfish from Ecuadorian Amazon

**DOI:** 10.1371/journal.pone.0287120

**Published:** 2023-07-12

**Authors:** Junior Chuctaya, Oscar Akio Shibatta, Andrea C. Encalada, Karla S. Barragán, Maria de Lourdes Torres, Estefanía Rojas, Valeria Ochoa-Herrera, Juliano Ferrer

**Affiliations:** 1 Programa de Pós-Graduação em Biologia Animal, Departamento de Zoologia, Universidade Federal do Rio Grande do Sul, Porto Alegre, Rio Grande do Sul, Brazil; 2 AQUAREC, Laboratorio de Biología y Genética Molecular, Instituto de Investigaciones de la Amazonia Peruana, Iquitos, Loreto, Peru; 3 Federal University of Uberlândia, Uberlândia, Minas Gerais, Brazil; 4 Museu de Zoologia, Departamento de Biologia Animal e Vegetal, Centro de Ciências Biológicas, Universidade Estadual de Londrina, Londrina, Paraná, Brazil; 5 Instituto BIOSFERA, Colegio de Ciencias Biológicas y Ambientales, Universidad San Francisco de Quito, Quito, Ecuador; 6 Laboratorio Biotecnología Vegetal, Colegio de Ciencias Biológicas y Ambientales, Universidad San Francisco de Quito, Quito, Ecuador; 7 Escuela de Ingeniería, Ciencia y Tecnología, Universidad del Rosario, Bogotá, Colombia; Fundacion Miguel Lillo, ARGENTINA

## Abstract

*Rhyacoglanis pulcher* is a rare Neotropical rheophilic bumblebee catfish known only from the type locality in the Cis-Andean Amazon region, Ecuador, and the type-species of the genus. So far, the three syntypes collected in 1880 were the only specimens unambiguously associated to the name *R*. *pulcher* available in scientific collections. Recently, a specimen was discovered in a fast-flowing stretch of the Villano river, a tributary of the Curaray river, Napo river basin, Ecuador, representing a new record after nearly 140 years. Here, we present this new record, identified by morphology, provide the DNA barcode sequence of the specimen, and propose why the species of *Rhyacoglanis* are scarce in zoological collections. Additionally, we discuss the intraspecific variation in the color pattern observed in *R*. *pulcher*.

## Introduction

The Pseudopimelodidae family is widely distributed in South America and Panama; contains two subfamilies, seven genera, and 55 valid species, of which 16 species were described in the last ten years [1–5]. Pseudopimelodids can be recognized externally by the wide mouth, small eyes without a free orbital margin, pectoral-fin spine serrated anteriorly and posteriorly, short barbels, and the coloration composed of variable patterns of dark brown blotches [6].

*Rhyacoglanis pulcher* (Boulenger, 1887) was originally described as “*Pimelodus* (*Pseudopimelodus*) *pulcher*” based on three syntypes collected in Canelos, upper Amazon river basin, Ecuador, in 1880, currently housed at the Natural History Museum (London, U.K.). Shibatta & Vari [1] redescribed and transferred the species to their newly proposed genus *Rhyacoglanis* based on a review of the type material and information from Boulenger [7]. *Rhyacoglanis* was proposed by Shibatta & Vari [1] to allocate five species based on the following characters: dorsal and lateral surfaces of head grey with a light blotch on the cheek; dark stripe along the midline of upper and lower caudal-fin lobes confluent with a dark caudal peduncle blotch; and 30–35 total vertebrae. Shibatta & Vari [1] designated *Rhyacoglanis pulcher* as the type-species of the genus and highlighted the absence of additional lots in scientific collections, suggesting that the species might be rare in the wild, even though they also assumed that it probably has a broader distribution in the Amazon basin.

Recently, a pseudopimelodid collected in the Villano river, a tributary of the Curaray river, Napo river basin in Ecuador, represents a new record of *Rhyacoglanis pulcher* after nearly 140 years of sampling gap. Here, we report on a new specimen of *R*. *pulcher* providing its DNA barcode sequence (cytochrome c oxidase subunit I), notes on habitat, and the first images of the species in life. We also discuss the color pattern variation observed in *R*. *pulcher* until now unexplored and investigate the reasons that make the *Rhyacoglanis*’ species scarce in scientific collections.

## Material and methods

### Ethical statement

Fish sampling was carried out in three regions of the Curaray river basin, Napo drainage, Ecuador, according to the following collection and genetic use permissions number 019-2018-IC-FAU-DNB, MAE-DNB-CM-2018-0106 issued by Ministerio del Ambiente of Ecuador, respectively as part of the project "Diversity and ecology of lotic ecosystems along elevation gradients, part of "Descubre Napo, NUNA Project” financed by Moore Foundation and by Collaboration Grant of Universidad San Francisco de Quito. The specimen collected was photographed *in vivo* by J. Viera, posteriorly, was euthanized by overdose with clove oil to remove the right pectoral and pelvic fins including in 99% ethanol as sample tissue, and fixed the specimen in 10% formalin solution. The fish were deposited in the ichthyologic collection of Instituto Nacional de Biodiversidad, Quito, Ecuador (MECN-DP). No surgical or experimental procedures were performed with live fish.

### Morphological analyses

Counts, measurements, and coloration description followed Shibatta & Vari [1]. Body measurements are presented as a percentage of standard length (SL), and head measurements are represented as a percentage of head length (HL). The weight of the specimen was taken just after the collection. Two x-rays of the specimen (from dorsal and lateral views) were performed to analyze osteological characters. The pectoral-fin ray was drawn with a camera lucida attached to a stereomicroscope. Comparative material examined is cited in Shibatta & Vari [1]. Principal components analysis (PCA) was applied on a variance matrix of log-normalized morphometrics variables of combined samples of the syntypes (N = 3) of *Rhyacoglanis pulcher* and the Villano river specimen using Past Program v.2.17c [8]. This analysis was employed to test the consistency of morphometric data within *R*. *pulcher* by adding of the new specimen.

### Molecular analyses

Genomic DNA was extracted from 20mg of an ethanol-preserved tissue sample following the CTAB method described by Doyle & Doyle [9]. The mitochondrial gene Cytochrome c oxidase subunit 1 (COI) was amplified via PCR using the fish universal primer set FishF1 and FishR1 [10], and following the conditions described in Carvalho *et al*. [11]. The PCR product was bidirectional sequenced at Macrogen Inc. (South Korea) using a high throughput Applied Biosystems 3037 XL technology. The obtained nucleotide sequence of the specimen (GenBank accession number—OP223115) was compared with sequences of other pseudopimelodids available in the NCBI GenBank (S1 Table). Species identity of four sequences of Genbank were corrected after a morphological analysis of their vouchers: "*Pseudopimelodus pulcher*" (EU179812) for *Rhyacoglanis* sp. (a potential new species) and "*Pseudopimelodus mangurus*" (GU701444, GU701870, GU701557) for *Rhyacoglanis paranensis*.

Sequences were aligned using MUSCLE algorithm [12] implemented in the Geneious 8.1.4 [13] under default parameters. Phylogenetic relationships were inferred by Bayesian inference (BI) in Beast 2.6.2 [14] via CIPRES portal v3.3 [15] with a strict molecular clock and a Yule model, using the general time reversible model with among-site rate heterogeneity GTR + I + G [16] as the best-fit evolutionary model estimated in jModelTest2 on XSEDE program [17] via CIPRES portal v3.3. The species tree was based on 100 million MCMC steps and sampled every 10000 steps with the chain efficiency observed in TRACER 1.7.1 [18] with 10% burn-in used to verify convergence and ESS values (>600). Maximum clade credibility (MCC) trees were summarized in Tree Annotator v1.75 [19] and visualized using the FigTree v1.4.3 [20].

We applied three methods of species delimitation: a) the Assemble Species by Automatic Partitioning (ASAP; [21]) through the aligned sequences and using Kimura 2-parameter (K2P; [22]) as genetic distance calculation, as input file at the ASAP webserver (https://bioinfo.mnhn.fr/abi/public/asap/asapweb.html) under default parameters; b) the Generalized Mixed Yule Coalescent (GYMC: [23]) using the single threshold parameter at the GMYC webserver (https://species.h-its.org/gmyc/); and c) the Bayesian Poisson tree processes (bPTP; [24]) with 100000 generations (thinning = 100) and another parameter at default.

Following the DNA barcoding for freshwater fishes [25], the pair-wise divergences of Pseudopimelodidae sequences grouped by species and genus were estimated using the K2P model in MEGAX [26].

### Map preparation

The distribution map was made with the help of the software QGis 3.10 [27] and Base map is downloaded from the USGS National Map Viewer (open access), including information from Boulenger [7] and Shibatta & Vari [1].

## Results

### Species identity

**Fig 1:**
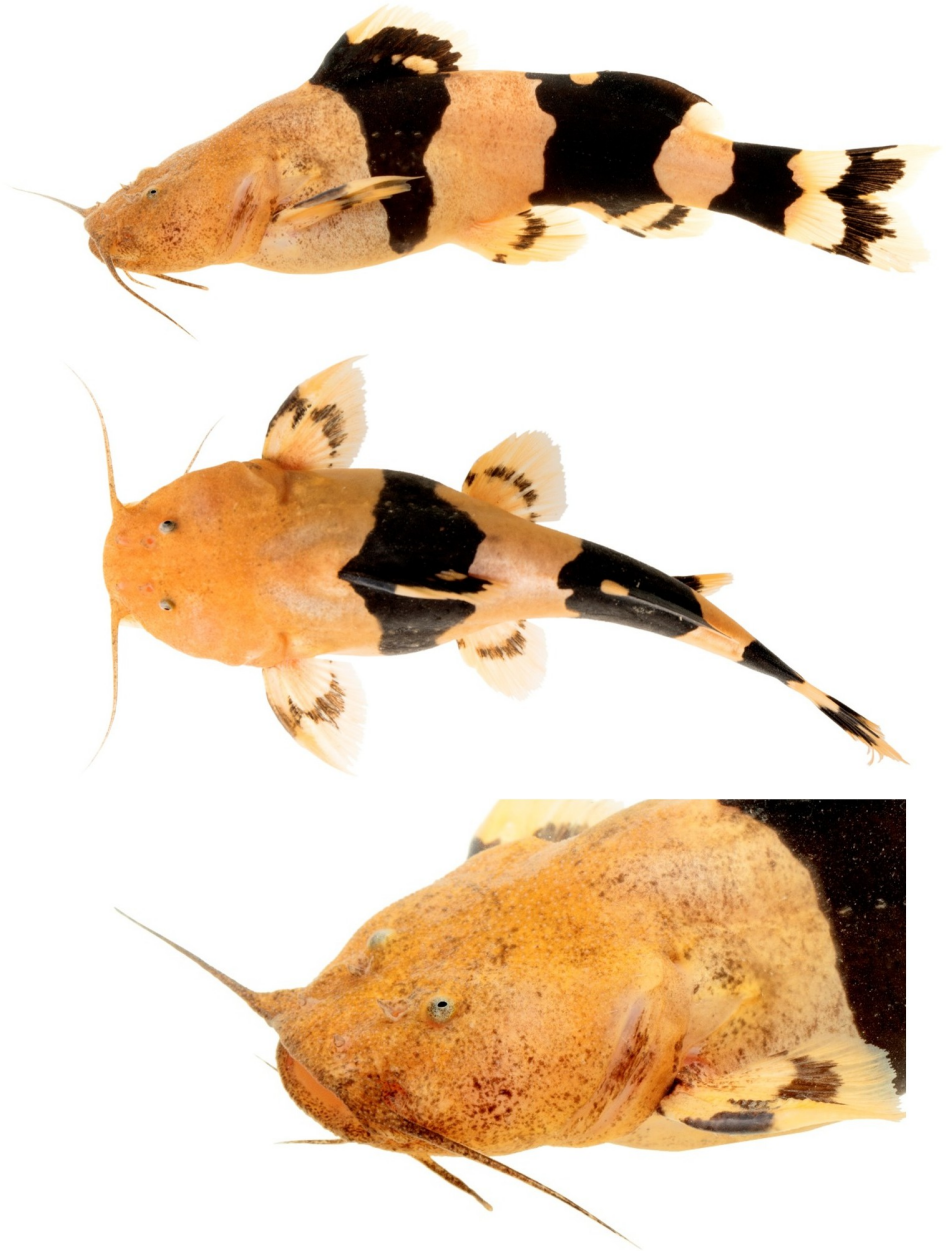
*Rhyacoglanis pulcher*, MECN-DP 4372, 82.1 mm SL, new record collected in Villano river, Napo river basin, Ecuador. Right pectoral and pelvic fins removed for tissue samples.

**Fig 2:**
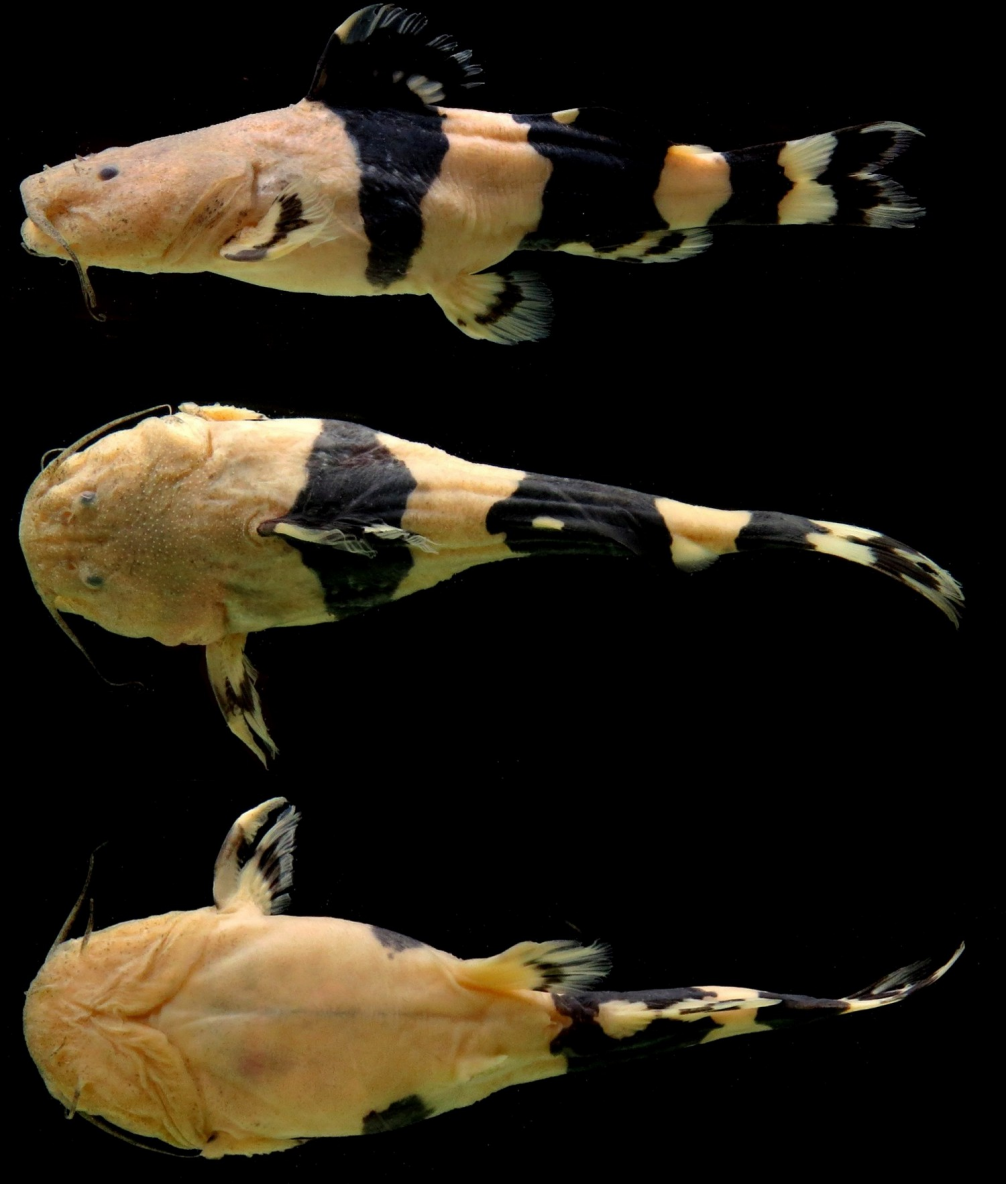
Color in life, *Rhyacoglanis pulcher*, MECN-DP 4372, 82.1 mm SL, new record collected in Villano river, Napo river basin, Ecuador. Dorsal view. (Photo: Jose Vieira).

**Fig 3:**
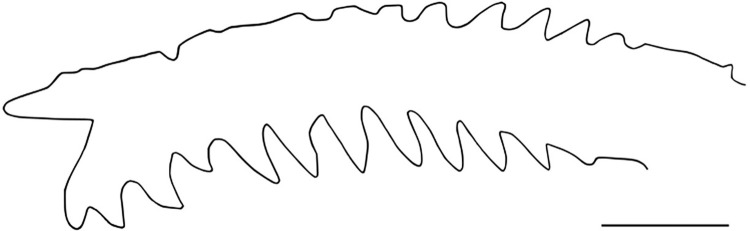
Ossified portion of left pectoral-fin spine of *Rhyacoglanis pulcher* from the Villano river, Napo river basin, Ecuador, MECN-DP 4372. Scale bar = 2.0 mm.

*Rhyacoglanis pulcher* (Pseudopimelodidae): MECN-DP 4372, 82.0mm SL and 17.2g weight (Figs 1–3). Taxon identity was based on the following characters observed in the specimen: body with three (subdorsal, subadipose, and caudal-peduncle) pronounced dark brown bands; subdorsal and subadipose bands well defined and not united; caudal-peduncle band uniformly dark; caudal-fin dark stripe W-shaped; caudal-fin lobes pointed (Figs 1 and 2); distal half of anterior margin of pectoral-fin spine serrae smaller than serrae of posterior margin (Fig 3); distance from the pelvic-fin origin to the anus (12.8); and 34 vertebrae (Table 1).

**Table 1:** Morphometric data of the new specimen collected in the Villano river and the three syntypes of *Rhyacoglanis pulcher* obtained from Shibatta and Vari (2017).

	MECN-DP 4372	Syn 1	Syn 2	Syn 3	Min-Max	Mean±SD
Standard length (mm)	82.6	68.5	67.4	58.5	58.5–82.6	69.2±10.0
**Percent of standard length**						
Head length	29.0	28.6	28.3	29.1	28.3–29.1	28.7±0.4
Pectoral-girdle width	28.5	27.8	26.5	27.6	26.5–28.5	27.6±0.8
Predorsal length	34.7	36.1	34.8	36.1	34.7–36.1	35.4±0.8
Dorsal-fin base length	17.1	15.6	16.8	15.8	15.6–17.1	16.3±0.7
Adipose-fin base length	24.2	20.9	15.1	19.3	15.1–24.2	19.8±3.8
Prepelvic length	53.8	51	51.4	52.8	51.0–53.8	52.2±1.3
Distance between pelvic and anal fins	20.0	24.5	24.4	26.7	20.0–26.7	23.9±2.8
Anal-fin base length	13.1	11.9	9.8	10.7	9.8–13.1	11.3±1.5
Caudal-peduncle length	18.1	17.8	18	15.6	15.6–18.1	17.4±1.2
Caudal-peduncle depth	9.9	16.9	18.1	17.9	9.8–10.1	9.9±0.1
Body depth	23.4	10	9.8	10.1	16.9–23.4	19.1±2.9
Pectoral-fin spine length	14.0	16.4	15.6	18.4	14.0–18.4	16.1±1.8
Dorsal-fin spine length	12.0	12.9	13.6	17.2	12.0–17.2	13.9±2.3
Pelvic-fin length	19.1	18.2	18.8	20	18.2–20.0	19.0±0.8
Postcleithral-process length	11.7	11.7	10.6	11	10.6–11.7	11.3±0.5
Distance between dorsal and pelvic fins	30.8	24.5	26.9	25	24.5–30.8	26.8±2.8
Distance between pelvic fins	13.8	13.1	14.2	13.5	13.1–14.2	13.6±0.5
Distance between pelvic fin and anus	12.8	12.9	11.1	14	11.1–14.0	12.7±1.2
Distance between anus and anal fin	8.8	15.2	13.8	14.4	8.8–15.2	13.0±2.9
**Percent of head length**						
Eye diameter	9	6	8	6	6–9	7±1.1
Interorbital distance	31	30	31	36	30–36	32±2.8
Snout length	36	37	36	39	36–39	37±1.7
Mouth width	66	54	48	63	48–66	58±8.1
Head depth	12	34	35	33	10–12	10±1.3
Maxillary-barbel length	80	71	71	80	71–80	75±5.1
Distance between anterior and posterior nostrils	15	18	19	17	15–19	17±1.6
Distance between posterior nostril and eye	9	6	8	6	6–9	7±1.6
Distance between posterior nostrils	20	18	24	24	18–24	21±2.7
**Counts**						
Dorsal-fin rays	I,6	I,6				
Pectoral-fin rays	I,6	I,6				
Pelvic-fin rays	i,5	i,5				
Anal-fin rays	iii,6	iii,6				
Caudal-fin rays	i,6,6,ii	i,6,6,ii				
Gill rakers	2,1,6	2,1,6				
Vertebrae	34	34				
Ribs	8	8				

### Collection station

**Fig 4:**
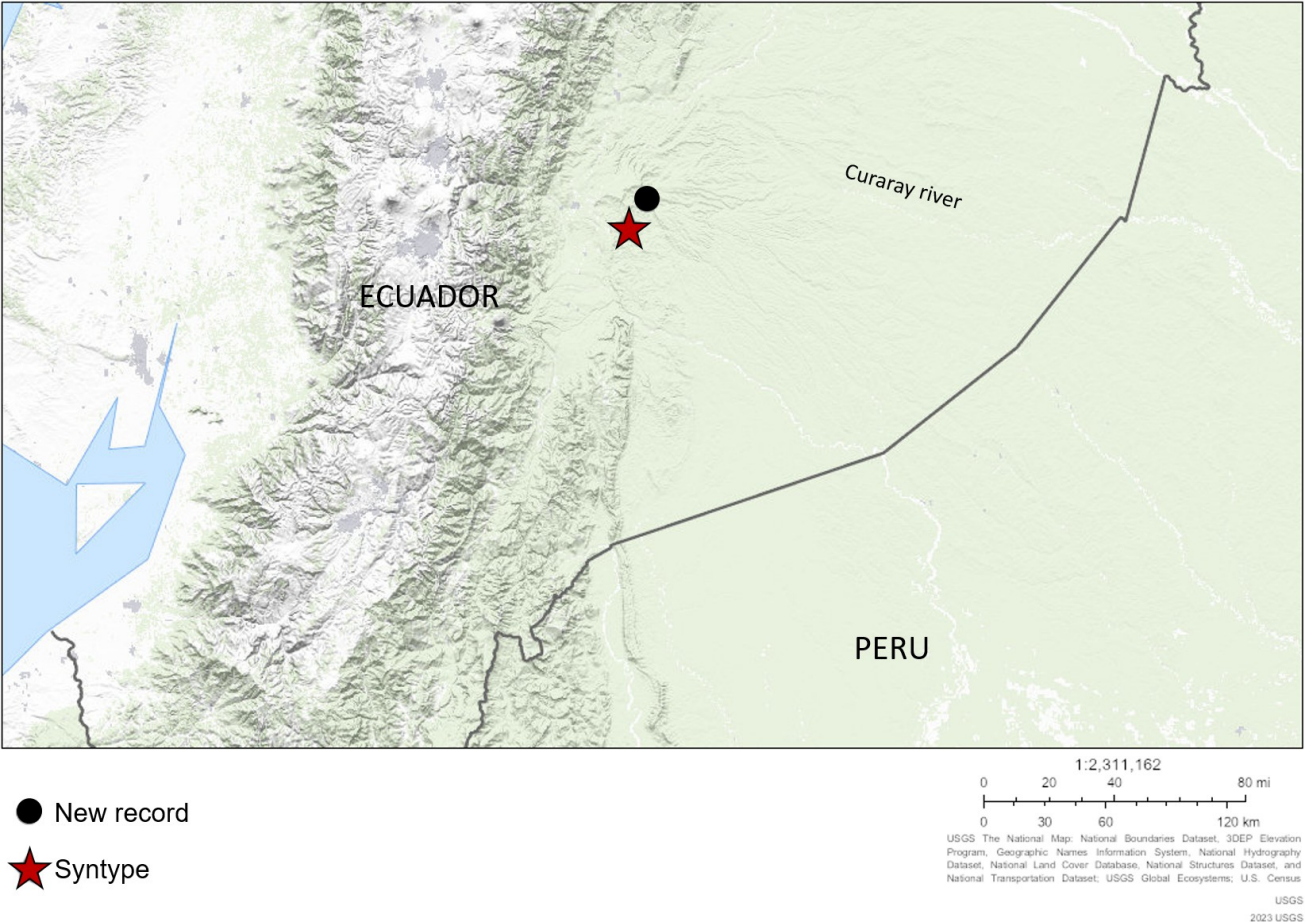
Geographical distribution of *Rhyacoglanis pulcher* in the Amazon basin based on examined specimens. Red star: type locality, black dot: the new record for the species. Base map is downloaded from the USGS National Map Viewer (open access) at http://viewer.nationalmap.gov/viewer/.

Villano river, a tributary of the Curaray river, Napo river basin, upper Amazon basin, 1°27.914’S, 77°40.762’W, at 473 m of altitude, collected by J. Chuctaya, K. Barragan, J. Vieira, November 14, 2018 (Fig 4).

### Habitat notes

**Fig 5:**
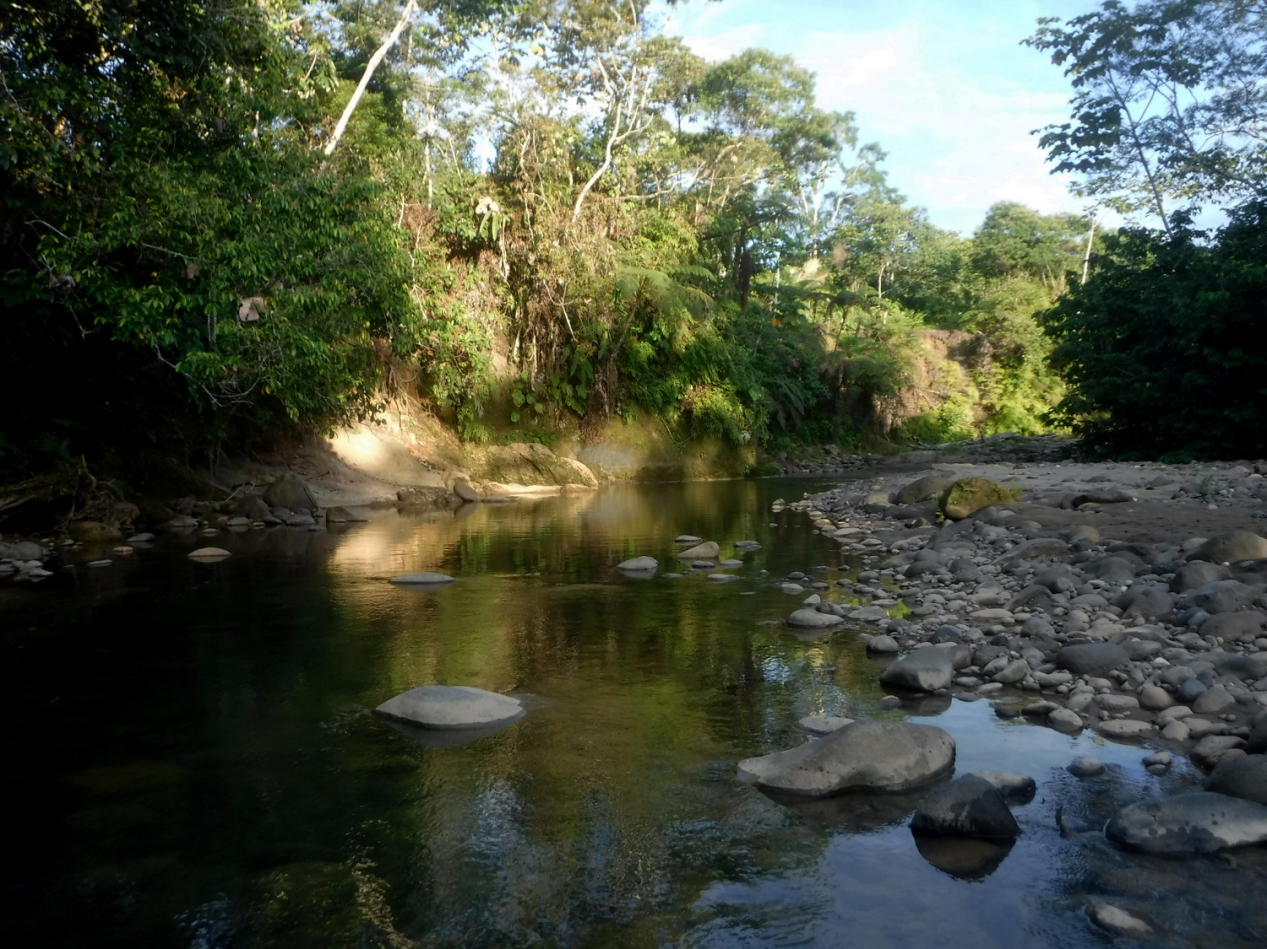
Collection station of *Rhyacoglanis pulcher* in the Villano river, tributary of Curaray river, Napo river basin, Ecuador.

The Villano river is a typical white-water Andean-Amazon piedmont river characterized by fast-flowing rapids with white water and sparse pools [28], with approximately 8–10 m of width. The stream bottom was rocky with 30% of boulders, 30% of cobbles, 20% of pebbles, 10% of gravel, and 10% of sand. The riparian vegetation is a lowland tropical rain forest with many trees including *Cecropia* spp., *Zygia* spp and *Inga* spp. The river is not polluted. However, the riparian vegetation is not intact, and some erosion has been coming from the banks. Small groups of indigenous and mestizo communities live close to the river and use it for domestic purposes and transportation. During our sampling, the water temperature was 28.1°C, pH was 8.25, conductivity was 128.7 μS/cm, and dissolved oxygen was 6.65 mg/L, with an estimated flow discharge of 4633.2 L/s. (Fig 5).

### Multivariate morphometric analysis

**Fig 6:**
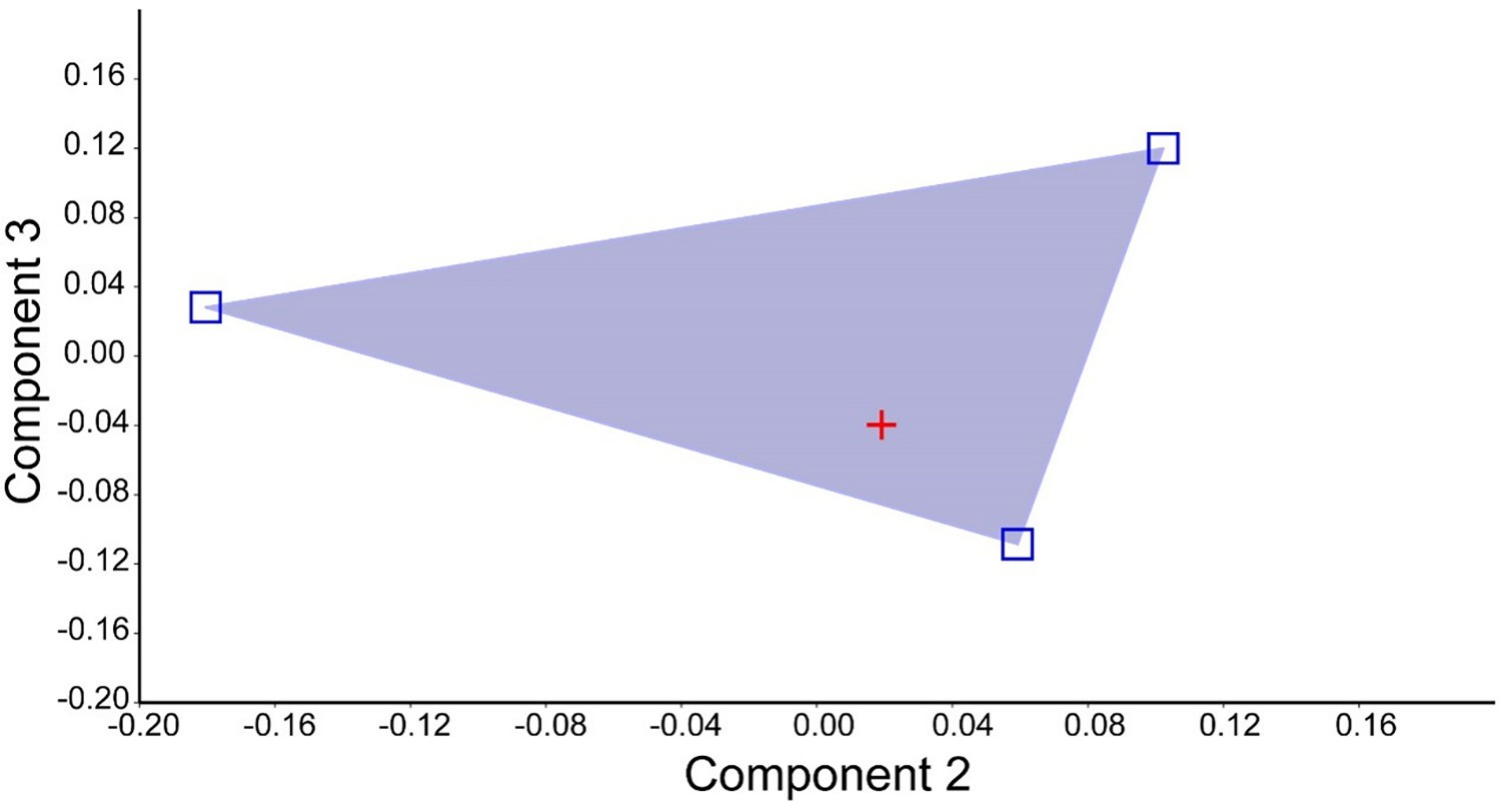
Scatterplot of individual scores of the syntypes of *Rhyacoglanis pulcher* syntypes (blue squares) and the specimen from Villano river (red cross) on the second and third principal components axis.

The first axis of the PCA retained 86.4% of data total variance, and all loadings of variables are positive, allowing interpretation as the representative of size (Table 2). The second and third axis of the PCA that retained 8.5% and 5.2 of the total variance, respectively (Fig 6: note that the specimen from Napo consistently groups with the three syntypes of *R*. *pulcher*). Additional information on morphometric and meristic are presented in Table 1, and loadings of variables in the first three principal components axis in Table 2.

**Table 2:** Variables loadings on the first two principal components axis of the syntypes of *Rhyacoglanis pulcher* and the specimen from the Villano river.

	Axis 1	Axis 2	Axis 3
Standard length	0,1501	-0,02636	0,1402
Head length	0,1531	0,005214	0,1008
Eye diameter	0,2903	-0,2079	-0,06843
Interorbital wdth	0,1042	0,09086	-0,2035
Snout length	0,1223	0,09142	-0,01182
Mouth width	0,2205	0,3269	-0,305
Pectoral girdle width	0,17	0,05516	0,1207
Predorsal distance	0,1335	0,02523	0,1482
Dorsal-fin base length	0,1848	-0,13	0,08572
Adipose-fin base length	0,2866	0,4964	0,04406
Prepelvic length	0,1661	-0,009878	0,05182
Pelvic fin to anal fin distance	0,01771	-0,009193	0,1174
Anal-fin base length	0,2559	0,2612	0,1678
Caudal peduncle length	0,2007	-0,1141	0,3587
Head depth	0,2736	-0,03775	0,006016
Body depth	0,2937	-0,05524	-0,1147
Caudal peduncle depth	0,1439	0,01207	0,1155
Pectoral-fin spine length	0,03695	0,09693	0,01553
Dorsal-fin spine length	0,01397	0,03978	-0,2661
Maxillary barbel length	0,1742	0,09506	-0,1646
Pelvic fin length	0,1408	-0,0179	-0,03293
Anterior to posterior nostrils distance	0,08099	-0,19	0,2446
Posterior nostril to eye distance	0,3114	-0,4891	-0,1639
Posterior nostrils distance	0,09554	-0,2612	-0,2811
Postcleithral process length	0,1819	0,09477	0,1933
Dorsal fin to pelvic fin distance	0,2529	-0,1303	0,01396
Pelvic fins distance	0,1587	-0,1397	0,08957
Pelvic fin to anus distance	0,1365	0,2648	-0,05876
Anus to anal fin distance	-0,1076	0,01309	0,5209
% variance	86,364	8,4736	5,1623

### Coloration in life

Body and head ground color pale orange, scattered with tiny reddish-brown spots (Fig 2). Body with three wide black transversal bands; subdorsal, subadipose, and caudal-peduncle. Subdorsal band nearly triangular, base dorsally, point ventrally, extending from dorsal fin towards to lateral surface of body; ventrally interrupted. Subadipose and caudal-peduncle bands circulating entire body. Subadipose band wider than subdorsal and caudal-peduncle bands; nearly rectangular with concave anterior and posterior margins; from adipose fin over lateral surface of body to anal-fin base. Caudal-peduncle band nearly crown-shaped, on caudal peduncle rear portion. Pectoral fin bright orange; transversal stripe reddish-brown, not continuous in middle portion, decreasing in width distally. Dorsal fin transversal stripe wide, reddish-brown; distal margin bright orange to hyaline; distal base elliptical area bright orange. Pelvic fin bright orange; transversal stripe reddish-brown not continuous in middle portion. Anal fin bright orange; two transversal stripes reddish-brown not continuous in base and middle portion. Caudal fin bright orange; transversal band reddish-brown in middle portion, confluent to caudal-peduncle band medially.

### Coloration in alcohol

**Fig 7:**
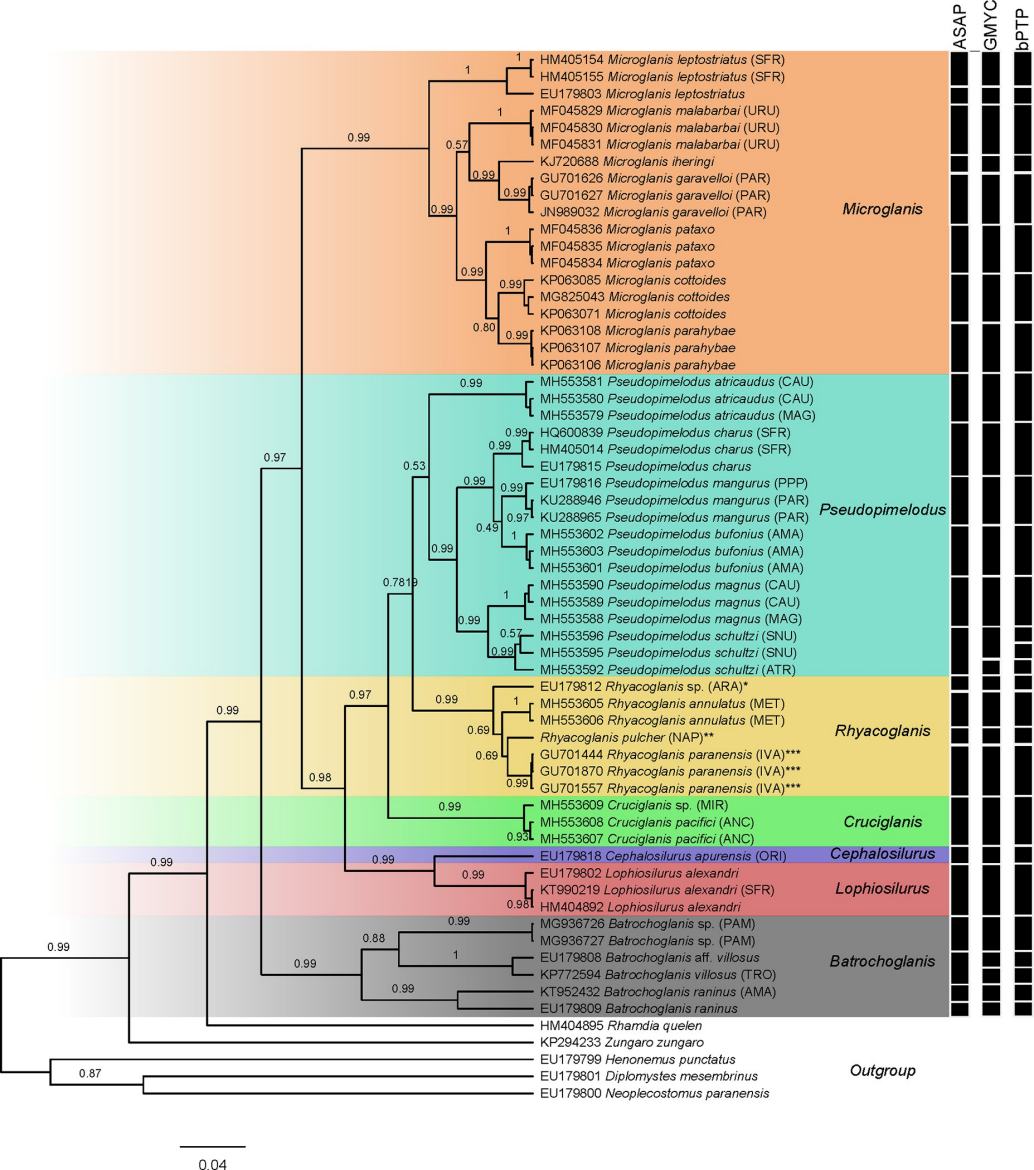
Syntypes of *Rhyacoglanis pulcher*, A: BMNH 1880.12.8.105-107c, 58.5 mm SL; B: BMNH 1880.12.8.105-107a, 67.4 mm SL; C: BMNH 1880.12.8.105-107b, 68.5 mm SL, from Canelos, Ecuador.

Coloration in alcohol similar but with ground color pale yellow and lateral bands black (Fig 1). Syntypes with lateral bands faded or orange-brown over grayish background (Fig 7).

### Molecular analysis

**Fig 8:**
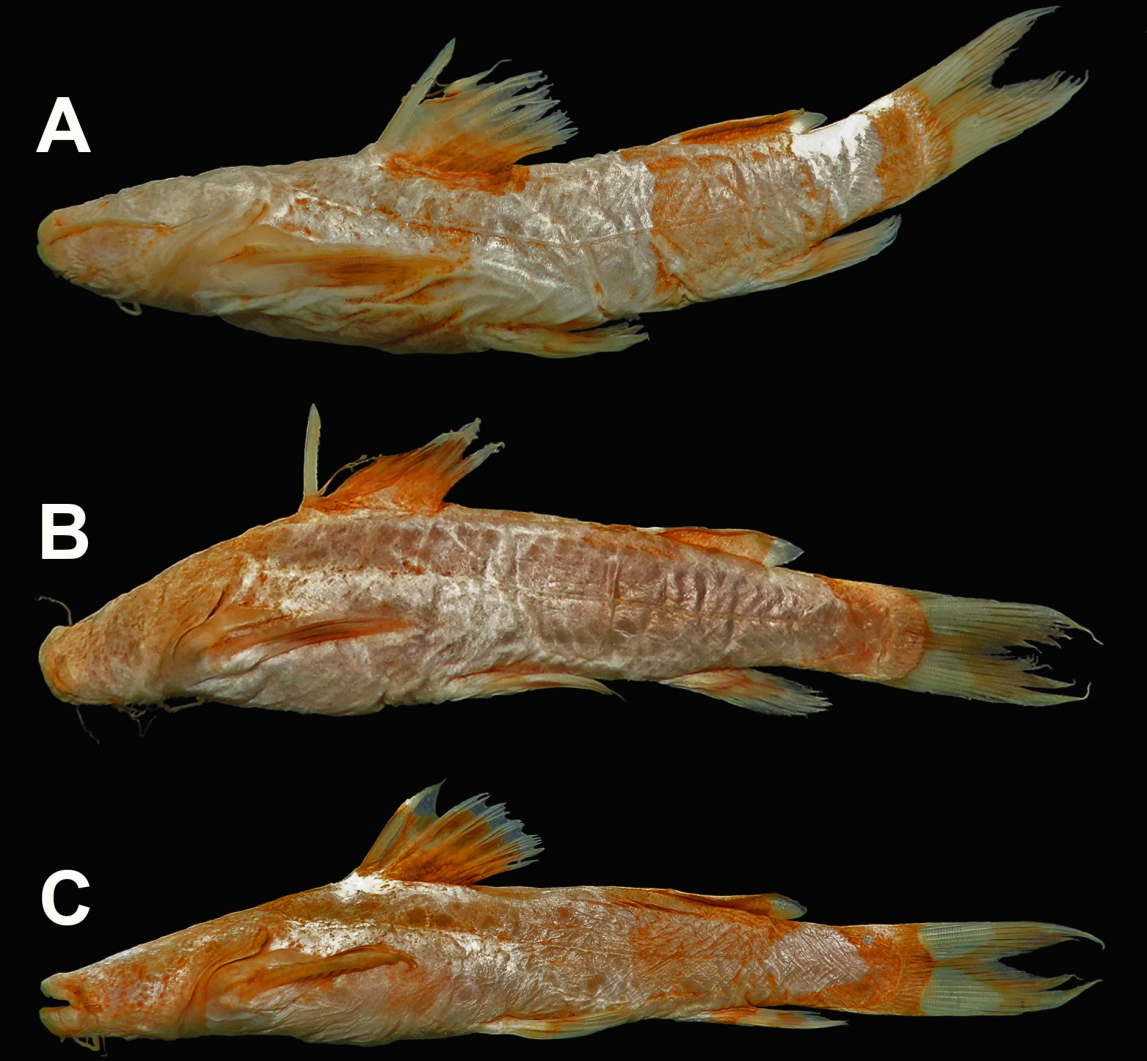
Species delimitation analyses based on sequences of Cytochrome c oxidase I (COI) sequences, using Assemble Species by Automatic Partitioning (ASAP), Poisson Tree Processes (PTP), and General Mixed Yule Coalescent (GMYC) methods. Bayesian (posterior probability, PP; above) support value for each node in the Bayesian phylogenetic COI tree. (*) sequences identity corrected to *Rhyacoglanis* sp.; (**) sequence of *R*. *pulcher* from Napo river basin, (***) sequences identity corrected corrected to *R*. *paranaensis*. AMA = Amazon river, ANC = Anchicava river, ARA = Das Mortes river, Araguaia river basin, ATR = Atrato river, CAU = Cauca river, IVA = Ivaí river, MAG = Magdalena river, MET = Meta river, MIR = Mira river, ORI = Orinoco river, PAM = Panamá, PAR = Paraná river, PPP = Paranapanema river, SFR = San Francisco river, SNU = Sinú river, TRO = Trombetasriver, and URU = Albino stream, Uruguay river basin.

The phylogenetic tree resulted from Bayesian inference (Fig 8) recovered all genera as monophyletic with high posterior probability values (PP). The specimen of *Rhyacoglanis pulcher* from Napo river basin was recovered as sister-group of *R*. *paranensis* being delimited as a single unit by the three methods of species delimitation (Fig 6; ASAP, GMYC and bPTP). Species delimitation using three methods returned slightly different results. ASAP suggests 25 groups within Pseudopimelodidae, GMYC suggests 27 groups and the bPTP suggests 28 species.

Genetic distance according to Kimura 2-parameter was relatively high between genera (Table 3), being the shortest distance between *Rhyacoglanis* and *Pseudopimelodus* (9.11) and the greatest distance between *Batrochoglanis* and *Cephalosilurus* (19.9). Among congeners, *Rhyacoglanis pulcher* presents the highest genetic distance from *R*. *annulatus* (3.9) and the lowest from *R*. *paranensis* (2.5) (Table 4).

**Table 3:** Estimates of evolutionary divergence over sequence Pairs between groups of genera. Values refer to the genetic distance based on COI sequences.

	Genus	1	2	3	4	5	6
1	*Pseudopimelodus*						
2	*Lophiosilurus*	13.26					
3	*Microglanis*	15.55	17.48				
4	*Rhyacoglanis*	9.11	12.86	15.59			
5	*Cephalosilurus*	15.65	10.61	17.46	14.14		
6	*Batrochoglanis*	17.35	18.54	16.53	16.30	19.92	
7	*Cruciglanis*	11.17	15.45	15.24	12.47	14.58	17.08

**Table 4:** Estimates of evolutionary divergence over sequence pairs between groups of species. Values refer to the genetic distance based on COI sequences.

	Species	1	2	3	4	5	6	7	8	9	10	11	12	13	14	15	16	17	18	19	20	21	22
1	*Pseudopimelodus charus*																						
2	*Lophiosilurus alexandri*	12.3																					
3	*Microglanis leptostriatus*	14.6	16.9																				
4	*Rhyacoglanis* sp.	9.5	12.6	13.7																			
5	*Pseudopimelodus mangurus*	3.5	11.8	13.0	9.2																		
6	*Cephalosilurus apurensis*	14.8	10.6	15.8	14.2	14.8																	
7	*Rhyacoglanis paranaensis*	10.5	14.6	14.5	3.8	9.1	15.2																
8	*Microglanis garavelloi*	16.3	17.7	8.5	13.2	14.9	16.9	15.4															
9	*Microglanis iheringi*	18.5	18.8	10.4	17.0	16.6	19.6	18.8	4.0														
10	*Microglanis cottoides*	14.5	16.7	7.7	14.3	13.3	18.3	14.7	7.1	9.6													
11	*Microglanis parahybae*	14.3	18.3	9.2	14.6	14.6	18.3	15.2	6.8	9.7	3.9												
12	*Batrochoglanis villosus*	17.2	17.2	15.6	15.7	17.2	19.1	17.1	15.9	16.9	15.4	17.1											
13	*Batrochoglanis raninus*	16.5	18.6	14.1	14.8	15.6	20.1	17.1	15.2	16.0	15.1	17.0	13.3										
14	*Microglanis malabarbai*	14.1	16.8	8.1	14.0	13.8	17.0	14.4	5.0	8.2	6.0	6.3	15.8	16.5									
15	*Microglanis pataxo*	14.3	17.9	9.2	14.3	14.5	17.8	14.9	6.8	9.4	4.8	4.1	16.8	17.0	5.1								
16	*Batrochoglanis* sp.	16.2	19.8	18.5	13.9	16.4	20.6	15.9	17.3	18.6	17.8	18.1	12.0	13.3	16.9	16.7							
17	*Rhyacoglanis annulatus*	10.5	12.8	16.0	5.7	10.7	14.1	4.5	16.2	20.5	17.4	17.0	17.3	16.5	14.7	17.7	17.8						
18	*Rhyacoglanis pulcher*	8.1	13.2	14.6	3.5	8.4	14.2	2.5	15.3	18.6	14.4	14.9	16.7	16.3	14.0	14.3	14.9	3.9					
19	*Pseudopimelodus bufonius*	3.6	12.8	14.8	9.5	2.8	16.2	9.5	17.2	19.8	14.9	15.8	17.8	15.6	16.1	15.3	18.3	10.7	8.3				
20	*Pseudopimelodus schultzi*	6.2	12.4	15.1	10.4	6.7	17.0	10.1	18.0	20.6	15.9	17.8	19.3	18.6	15.0	15.7	18.3	10.6	8.9	6.0			
21	*Pseudopimelodus magnus*	6.4	14.6	16.6	8.9	6.8	15.9	9.1	17.4	21.0	14.8	17.1	19.3	17.9	15.9	16.2	18.2	10.4	7.8	6.5	4.5		
22	*Pseudopimelodus atricaudus*	7.5	14.4	13.7	11.8	8.3	15.7	11.6	17.0	19.2	14.0	17.3	17.9	15.6	16.3	15.8	18.4	12.1	8.9	8.5	10.2	9.8	
23	*Cruciglanis pacifici*	11.4	15.4	13.7	11.7	10.0	14.6	13.2	17.0	20.5	13.4	15.0	17.9	15.9	15.3	15.2	17.4	13.3	11.5	10.8	11.9	9.8	11.1

## Discussion

The pimelodid recorded was confirmed by a set of external and internal characters as *Rhyacoglanis pulcher* (see results) the longest specimen known of the species, with 82.0 mm SL (Figs 1 and 2). Boulenger [7] described *Pimelodus (Pseudopimelodus) pulcher* based on three syntypes with 58.5 to 68.5 mm SL. A multivariate morphometric analysis including these four specimens found the non-type inserted within the syntypes variation (Fig 6). Additionally, the specimen of *R*. *pulcher* was recovered as a taxonomic unit in the three methods of species delimitation applied (Fig 7; ASAP, GMYC and bPTP).

The new specimen of *Rhyacoglanis pulcher* recently collected and photographed in life provides important data related to coloration in addition to the two black-and-white illustrations provided by Boulenger ([7]; Fig 1) and the redescription of Shibatta & Vari [1]. The non-type (MECN-DP 4372; Figs 1 and 2) and the three syntypes (BMNH 1880.12.8.105–107; Fig 8) have a similar color pattern with few variations in the shape of the lateral dark bands. The three lateral bands of one syntype are strongly confluent dorsally and ventrally forming an ellipsoid light blotch (Fig 8C), a condition not presented in the non-type (Figs 1 and 2) as well as in another syntype, which has only the ventral portion of the subadipose and caudal-peduncle dark bands in contact (Fig 8A). The third syntype (Fig 8B) shows the caudal-peduncle dark band confluent dorsally and ventrally, but the subadipose is poorly visible. Additionally, the non-type has the midline portions of caudal fin and caudal-peduncle bands confluent, but not in contact (Figs 1 and 2; *vs*. bands confluent and contacting each other in all syntypes; Fig 8), and the dark stripes of the pectoral and pelvic fins not continuous (*vs*. continuous in the syntypes). Intraspecific variation in the coloration was also observed in the congeners *Rhycoglanis epiblepsis* and *R*. *seminiger* by Shibatta & Vari [1].

This study provides the first COI sequence of *Rhyacoglanis pulcher* associated with a voucher properly identified. The supposed COI records for "*Rhyacoglanis pulcher*" used in some studies [29–31], and available in Genbank (EU179812, LBP1567, Das Mortes river) actually represent a single sequence, which show a genetic distance of 3.5% compared with the specimen herein analyzed (Table 4). Thereby, we corrected the “Das Mortes river” COI record to *Rhyacoglanis* sp. in our molecular analyses (Fig 7), which is a potentially undescribed species and highlighted the importance of checking the identity of voucher before including them in any study. Our phylogenetic analysis performed with COI found *R*. *pulcher* as sister-group of *R*. *paranensis* whereas the morphological approach of Shibatta & Vari [1] shows a close relationship with *R*. *seminiger*. Both results differ from the molecular phylogeny using ultra-conserved elements performed by Silva *et al*. [4], in which a specimen from Maranõn river (Peru) identified as *R*. *pulcher* is the first to diverge within the genus. However, Silva et al. [4] did not provide photographs of the voucher or details in how they proceeded the identification. Consequently, we recommend revaluating the specimen identity in light of the present study before assuming it as *R*. *pulcher*.

So far, the three syntypes from Canelos (Ecuador) were the only specimens unambiguously belonging to *R*. *pulcher* preserved in scientific collections. Consequently, Shibatta & Vari [1] classified the species as deficient in data (DD) according to the IUCN guidelines, a category applied when there is inadequate information to evaluate the risk of extinction based on the distribution or population status of a taxon, but the available information indicates that the species can be threatened [32]. In fact, the new record increased the distribution range of *R*. *pulcher*. However, we consider that a single specimen from new locality, relatively near to the type locality and where was observed some degree of erosion in the river and deforestation in the banks, is insufficient to change the DD category.

Shibatta & Vari [1] suggested that *Rhyacoglanis pulcher*, along with *R*. *annulatus* and *R*. *seminiger*, may be rare due to their records scarcity, although they also assumed that the species probably have a wider distribution than currently known. *Rhyacoglanis annulatus* (Orinoco river basin, Venezuela) and *R*. *seminiger* (Tapajós river basin, Brazil) are known only from their type material: two specimens from one locality each and 16 specimens from three localities, respectively. Additionally, *R*. *epiblepsis* (Madeira-Mamoré river basin, Bolivia) is known only from the type locality but was described with 116 paratypes. *Rhyacoglanis paranensis* is widely distribute in the upper Paraná river basin (Brazil), an exception within the genus. However, it is important to mention that the Paraná river basin is one of South America’ most inventoried in terms of freshwater fishes [33, 34].

The few records of species of *Rhyacoglanis* could be related to one or more of the following reasons: 1) lack of samplings, mainly in remote regions that are insufficiently inventoried, such as the western Amazon basin headwaters; 2) specific habitats occupied by the species, such as riffles and other rocky swift-flowing waters (Shibatta & Vari, 2017); 3) unappropriated fisheries methods employed in field works, which make the collection a challenging task; and 4) lack of comprehensive reviews of pseudopimelodids in scientific collections of South America. The specimen reported herein was collected in only one of 30 collection stations carried out in a field expedition to the Napo river basin, which clearly presents an underestimated species-rich of freshwater fishes [35]. Traditional fisheries methods, such as beach seines nets, gill nets, and hand nets, were not efficient in capturing the species, which was sampled exclusively with the aid of electrofishing in a river stretch with high water conductivity.

## Supporting information

S1 Table Sequences of pseudopimelodids generated in this study (in bold) and those download from GenBank used in the analyses. a: species previously identified as *Pseudopimelodus mangurus*; b: species previously identified as *Rhyacoglanis pulcher*; (*) indicates vouchers without locality information.
(DOCX)
