# Correction: Rediscovery of *Rhyacoglanis pulcher* (Boulenger, 1887) (Siluriformes: Pseudopimelodidae), a rare rheophilic bumblebee catfish from Ecuadorian Amazon

**DOI:** 10.1371/journal.pone.0307566

**Published:** 2024-07-18

**Authors:** Junior Chuctaya, Oscar Akio Shibatta, Andrea C. Encalada, Karla S. Barragán, Maria de Lourdes Torres, Estefanía Rojas, Valeria Ochoa-Herrera, Juliano Ferrer

The images for Figs 1 and 2 are incorrectly switched. The image that appears as Fig 1 should be Fig 2, and the image that appears as Fig 2 should be Fig 1. The images for Figs 7 and 8 are incorrectly switched. The image that appears as Fig 7 should be Fig 8, and the image that appears as Fig 8 should be Fig 7. The figure captions appear in the correct order.

**Fig 1:**
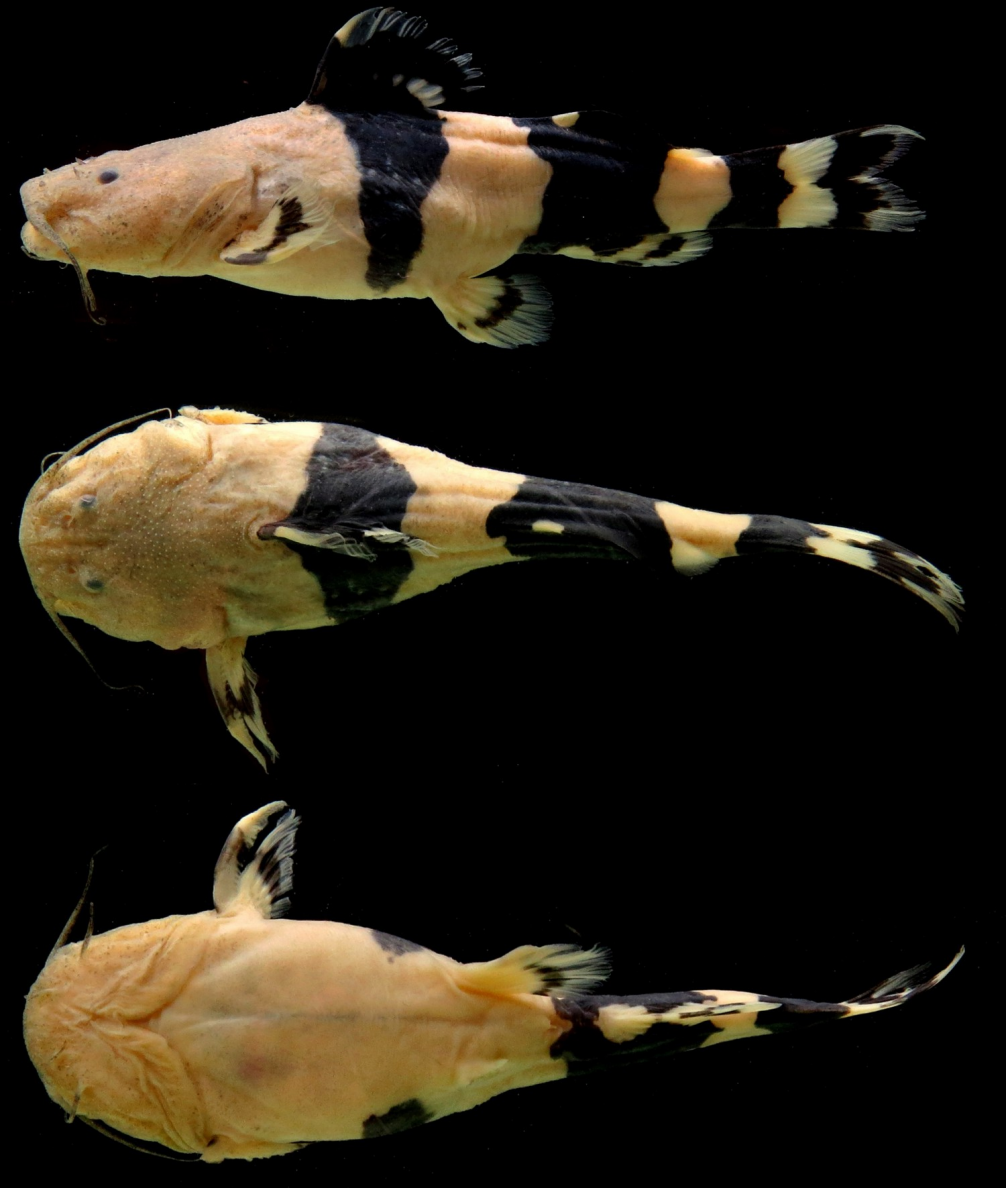
*Rhyacoglanis pulcher*, MECN-DP 4372, 82.1 mm SL, new record collected in Villano river, Napo river basin, Ecuador. Right pectoral and pelvic fins removed for tissue samples.

**Fig 2:**
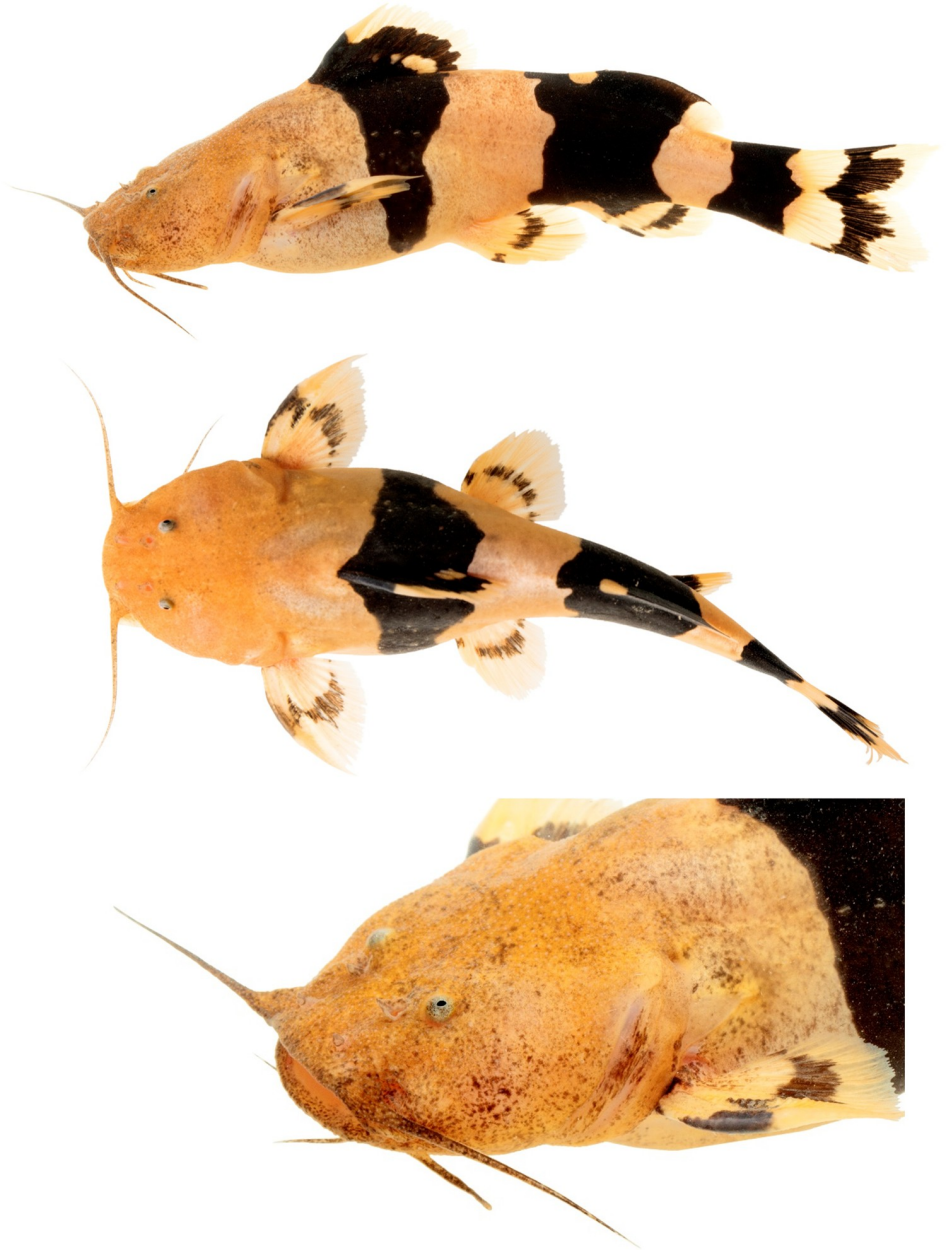
Color in life, *Rhyacoglanis pulcher*, MECN-DP 4372, 82.1 mm SL, new record collected in Villano river, Napo river basin, Ecuador. Dorsal view. (Photo: Jose Vieira).

**Fig 7:**
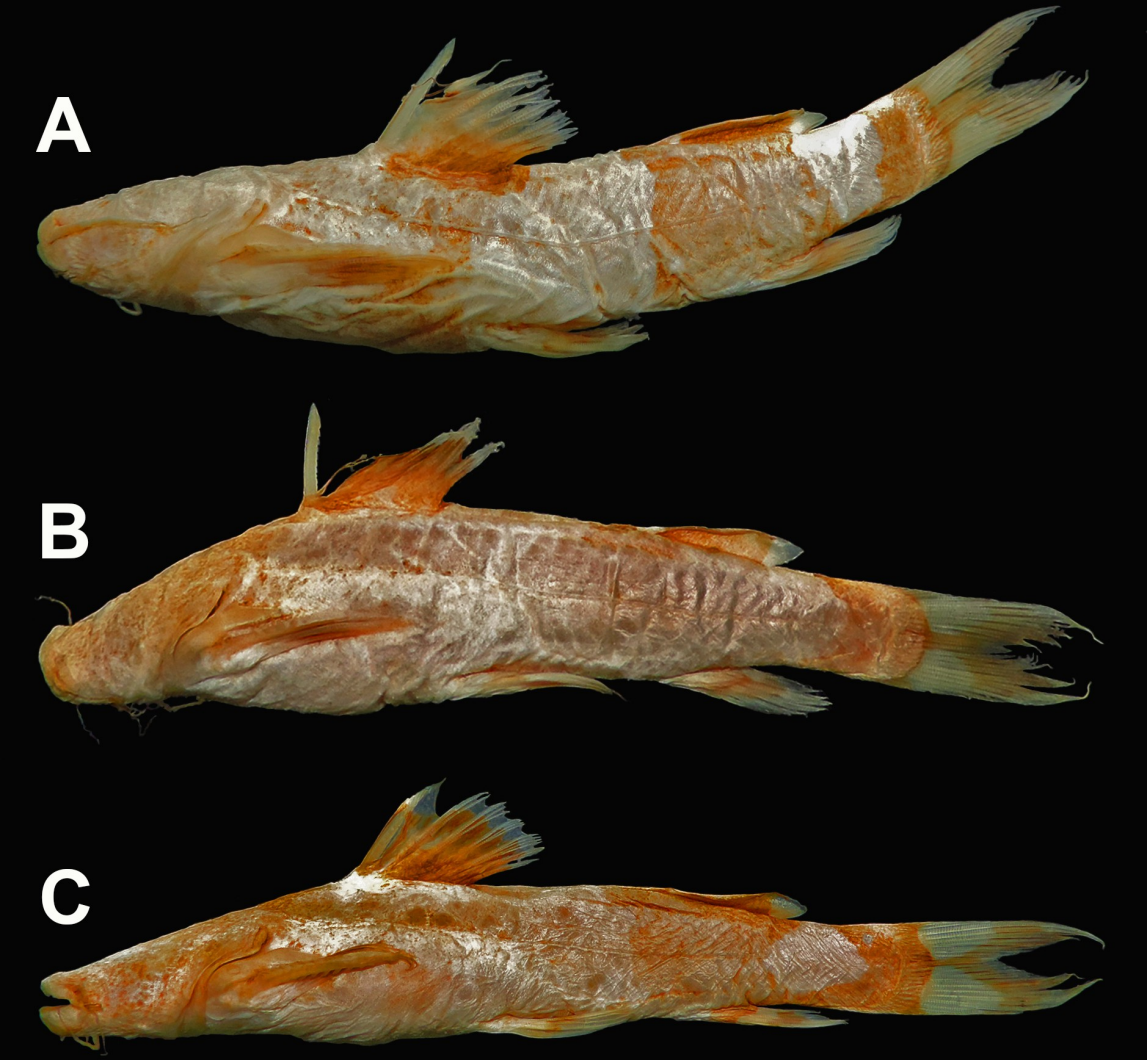
Syntypes of *Rhyacoglanis pulcher*, A: BMNH 1880.12.8.105-107c, 58.5 mm SL; B: BMNH 1880.12.8.105-107a, 67.4 mm SL; C: BMNH 1880.12.8.105-107b, 68.5 mm SL, from Canelos, Ecuador.

**Fig 8:**
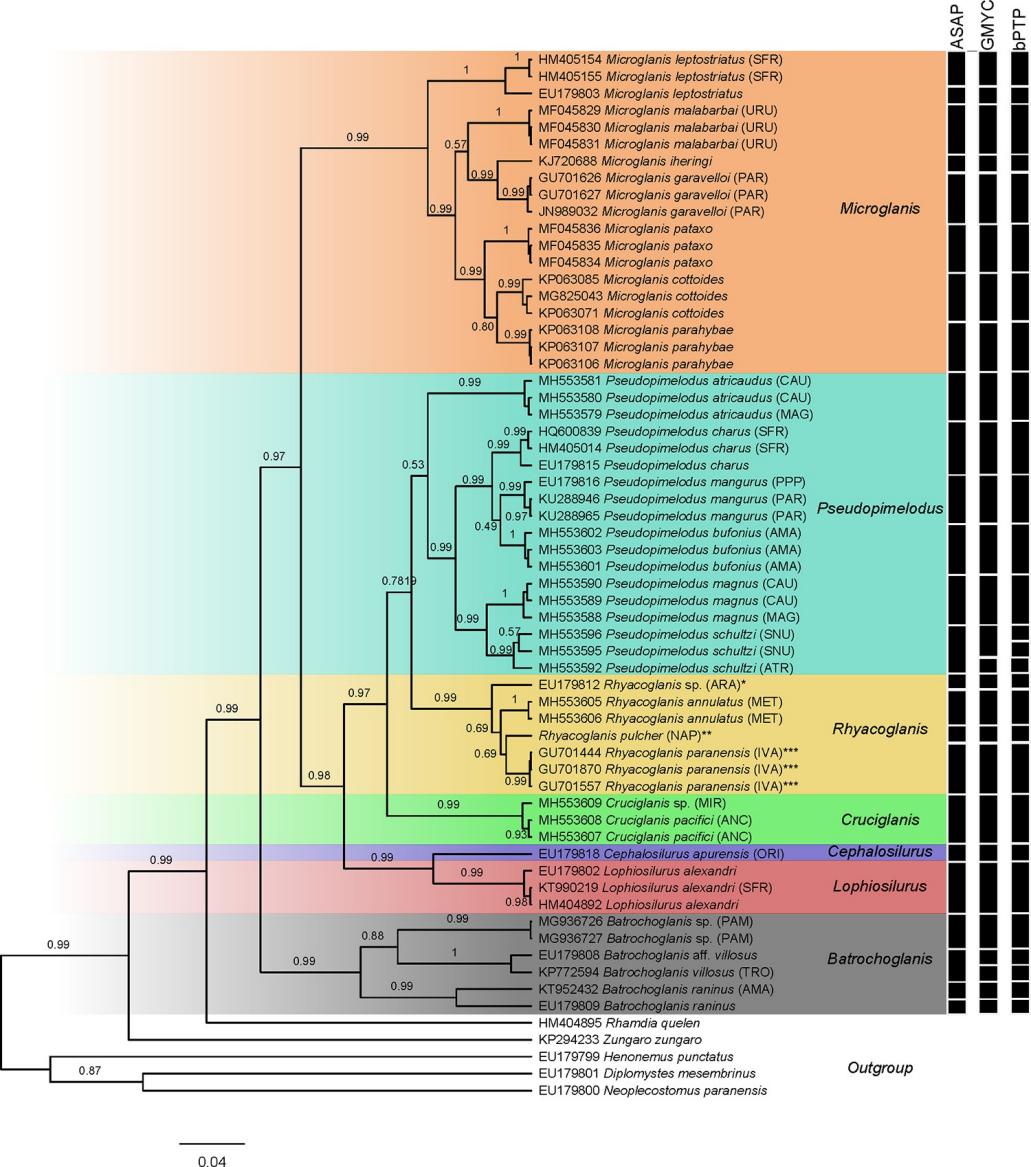
Species delimitation analyses based on sequences of Cytochrome c oxidase I (COI) sequences, using Assemble Species by Automatic Partitioning (ASAP), Poisson Tree Processes (PTP), and General Mixed Yule Coalescent (GMYC) methods. Bayesian (posterior probability, PP; above) support value for each node in the Bayesian phylogenetic COI tree. (*) sequences identity corrected to *Rhyacoglanis* sp.; (**) sequence of *R*. *pulcher* from Napo river basin, (***) sequences identity corrected corrected to *R*. *paranaensis*. AMA = Amazon river, ANC = Anchicava river, ARA = Das Mortes river, Araguaia river basin, ATR = Atrato river, CAU = Cauca river, IVA = Ivaí river, MAG = Magdalena river, MET = Meta river, MIR = Mira river, ORI = Orinoco river, PAM = Panamá, PAR = Paraná river, PPP = Paranapanema river, SFR = San Francisco river, SNU = Sinú river, TRO = Trombetasriver, and URU = Albino stream, Uruguay river basin.
