# Supplementary material for: Rediscovery of Rhyacoglanis pulcher (Boulenger, 1887) (Siluriformes: Pseudopimelodidae), a rare rheophilic bumblebee catfish from Ecuadorian Amazon
Source: PLoS One. 2023 Jul 12;18(7):e0287120. doi: 10.1371/journal.pone.0287120 (PMC10337946; doi:10.1371/journal.pone.0287120)
Supplement: S1 Table — a: species previously identified as Pseudopimelodus mangurus; b: species previously identified as Rhyacoglanis pulcher; (*) indicates vouchers without locality information. (DOCX) [file pone.0287120.s001.docx]

**S1 Table.** Sequences of pseudopimelodids generated in this study (in bold) and those download from GenBank used in the analyses. a: species previously identified as *Pseudopimelodus mangurus*; b: species previously identified as *Rhyacoglanis pulcher*; (*) indicates vouchers without locality information.

| **N** | **Gbank** | **species** | **Code** | **Locality** | **Reference** |
| --- | --- | --- | --- | --- | --- |
| 1 | EU179802 | *Lophiosilurus alexandri* | LBP-276 | * | Martinez et al. 2007 |
| 2 | HM404892 | *Lophiosilurus alexandri* | DCC-4235 | San Francisco River | Carvalho et al. 2011 |
| 3 | KT990219 | *Lophiosilurus alexandri* | complete genome | * | Resende et al. 2015 |
| 4 | EU179818 | *Cephalosilurus apurensis* | LBP-3034 | Orinoco River | Martinez et al. 2007 |
| 5 | HM405014 | *Pseudopimelodus charus* | DCC-13715 | San Francisco River | Carvalho et al. 2011 |
| 6 | HQ600839 | *Pseudopimelodus charus* | DCC-00657 | San Francisco River | Carvalho et al. 2011 |
| 7 | EU179815 | *Pseudopimelodus charus* | LBP-1967 | * | Martinez et al. 2007 |
| 8 | KU288965 | *Pseudopimelodus mangurus* | MGZV-P197 | Paraná River | Diaz et al. 2016 |
| 9 | KU288946 | *Pseudopimelodus mangurus* | MGZV-P197-3 | Paraná River | Diaz et al. 2016 |
| 10 | EU179816 | *Pseudopimelodus mangurus* | LBP-2168 | Paranapanema River | Martinez et al. 2007 |
| 11 | MH553601 | *Pseudopimelodus bufonius* | PSL2-H31 | Amazon River | Restrepo-Goméz et al. 2020 |
| 12 | MH553602 | *Pseudopimelodus bufonius* | PSL2-H32 | Amazon River | Restrepo-Goméz et al. 2020 |
| 13 | MH553603 | *Pseudopimelodus bufonius* | PSL2-H33 | Amazon River | Restrepo-Goméz et al. 2020 |
| 14 | MH553589 | *Pseudopimelodus magnus* | PSL5-H19 | Cauca River | Restrepo-Goméz et al. 2020 |
| 15 | MH553590 | *Pseudopimelodus magnus* | PSL5-H20 | Cauca River | Restrepo-Goméz et al. 2020 |
| 16 | MH553588 | *Pseudopimelodus magnus* | PSL5-H18 | Magdalena River | Restrepo-Goméz et al. 2020 |
| 17 | MH553595 | *Pseudopimelodus schultzi* | PSL4-H25 | Sinú River | Restrepo-Goméz et al. 2020 |
| 18 | MH553596 | *Pseudopimelodus schultzi* | PSL4-H26 | Sinú River | Restrepo-Goméz et al. 2020 |
| 19 | MH553592 | *Pseudopimelodus schultzi* | PSL4-H22 | Atrato River | Restrepo-Goméz et al. 2020 |
| 20 | MH553580 | *Pseudopimelodus atricaudus* | PSL1-H10 | Magdalena River | Restrepo-Goméz et al. 2020 |
| 21 | MH553579 | *Pseudopimelodus atricaudus* | PSL1-H9 | Cauca River | Restrepo-Goméz et al. 2020 |
| 22 | MH553581 | *Pseudopimelodus atricaudus* | PSL1-H11 | Cauca River | Restrepo-Goméz et al. 2020 |
| **23** | **OP223115** | ***Rhyacoglanis pulcher*** | **MECN-DP-4372** | **Napo River** | **This study** |
| 24 | GU701557 | *Rhyacoglanis paranenis*^a^ | LBP-37228 | Ivaí River | Pereira et al. 2013 |
| 25 | GU701870 | *Rhyacoglanis paranenis*^a^ | LBP-37227 | Ivaí River | Pereira et al. 2013 |
| 26 | GU701444 | *Rhyacoglanis paranenis*^a^ | LBP-37226 | Ivaí River | Pereira et al. 2013 |
| 27 | MH553605 | *Rhyacoglanis annulatus* | AOL-097 | Meta River | Restrepo-Goméz et al. 2020 |
| 28 | MH553606 | *Rhyacoglanis annulatus* | AOL-098 | Meta River | Restrepo-Goméz et al. 2020 |
| 29 | EU179812 | *Rhyacoglanis* sp.^b^ | LBP1567 | Das Mortes River | Martinez et al. 2007 |
| 30 | MH553609 | *Cruciglanis sp.* | AOL-024 | Mira River | Restrepo-Goméz et al. 2020 |
| 31 | MH553608 | *Cruciglanis pacifici* | AOL-023 | Anchicayá River | Restrepo-Goméz et al. 2020 |
| 32 | MH553607 | *Cruciglanis pacifici* | AOL-094 | Anchicayá River | Restrepo-Goméz et al. 2020 |
| 33 | HM405155 | *Microglanis leptostriatus* | DCC-00613 | San Francisco River | Carvalho et al. 2011 |
| 34 | HM405154 | *Microglanis leptostriatus* | DCC-00612 | San Francisco River | Carvalho et al. 2011 |
| 35 | EU179803 | *Microglanis leptostriatus* | LBP-335 | * | Martinez et al. 2007 |
| 36 | KP063107 | *Microglanis parahybae* | Mcott-49 | * | Souza-Shibatta et al. 2018 |
| 37 | KP063106 | *Microglanis parahybae* | Mcott-48 | * | Souza-Shibatta et al. 2018 |
| 38 | KP063108 | *Microglanis parahybae* | Mcott-50 | * | Souza-Shibatta et al. 2018 |
| 39 | MG825043 | *Microglanis cottoides* | MNLM-4982 | * | Buckup et al. 2018 |
| 40 | KP063071 | *Microglanis cottoides* | Mcott-13 | * | Souza-Shibatta et al. 2018 |
| 41 | KP063085 | *Microglanis cottoides* | Mcott-27 | * | Souza-Shibatta et al. 2018 |
| 42 | MF045836 | *Microglanis pataxo* | MZUEL-17780.3 | * | Souza-Shibatta et al. 2018 |
| 43 | MF045835 | *Microglanis pataxo* | MZUEL-17780.2 | * | Souza-Shibatta et al. 2018 |
| 44 | MF045834 | *Microglanis pataxo* | MZUEL-17780.1 | * | Souza-Shibatta et al. 2018 |
| 45 | MF045830 | *Microglanis malabarbai* | MZUEL-17059.2 | Albino stream, Uruguay basin | Souza-Shibatta et al. 2018 |
| 46 | MF045831 | *Microglanis malabarbai* | MZUEL-17059.3 | Albino stream, Uruguay basin | Souza-Shibatta et al. 2018 |
| 47 | MF045829 | *Microglanis malabarbai* | MZUEL-17059.1 | Albino stream, Uruguay basin | Souza-Shibatta et al. 2018 |
| 48 | KJ720688 | *Microglanis iheringi* | Kasc-2 | * | Premdass et al. 2014 |
| 49 | GU701627 | *Microglanis garavelloi* | LBP-22539 | Parana River | Pereira et al. 2013 |
| 50 | GU701626 | *Microglanis garavelloi* | LBP-22540 | Parana River | Pereira et al. 2013 |
| 51 | JN989032 | *Microglanis garavelloi* | LBPV-10562 | Parana River | Pereira et al. 2013 |
| 52 | KP772594 | *Batrochoglanis villosus* | INPA-43882 | Trombetas River | Collins et al. 2015 |
| 53 | EU179808 | *Batrochoglanis aff. villosus* | LBP-2347 | * | Martinez et al. 2007 |
| 54 | MG936727 | *Batrochoglanis sp. nov.* | stri-6745 | Panamá | Bermingham et al. 2018 |
| 55 | MG936726 | *Batrochoglanis sp. nov.* | stri-6758 | Panamá | Bermingham et al. 2018 |
| 56 | EU179809 | *Batrochoglanis raninus* | LBP-1777 | * | Martinez et al. 2007 |
| 57 | KT952432 | *Batrochoglanis raninus* | A19-UM2-IRD | Amazonas River | Garcia-Davila et al. 2015 |
| 58 | HM404895 | *Rhamdia quelen* | DCC-4326 | San Francisco River | Carvalho et al. 2011 |
| 59 | KP294233 | *Zungaro zungaro* | P-233 | * | Lima, 2014 |
| 60 | EU179799 | *Henonemus punctatus* | LBP-1125 | * | Martinez et al. 2007 |
| 61 | EU179801 | *Diplomystes mesembrinus* | LBP-449 | * | Martinez et al. 2007 |
| 62 | EU179800 | *Neoplecostomus paranensis* | LBP-709 | * | Martinez et al. 2007 |

**Reference**

Bermingham E, Reina RG, Sanjur O. Freshwater Fish of Panama. Submitted (FEB 2018) to the EMBL/GenBank/ DDBJ databases.2018.

Buckup PA, Jennings WB, Ferraro GA. New barcode primers. Submitted (JAN 2018) to the EMBL/GenBank/ DDBJ databases.2018.

Carvalho DC, Oliveira DA, Pompeu PS, Leal CG, Oliveira C, Hanner R. Deep barcode divergence in Brazilian freshwater fishes: the case of the São Francisco River basin. Mitochondrial Dna, 2011. 22(sup1), 80-86.

Collins RA, Ribeiro ED, Machado VN, Hrbek T, Farias IP. A preliminary inventory of the catfishes of the lower Rio Nhamundá, Brazil (Ostariophysi, Siluriformes). Biodivers. Data J. 2015. 3, e4162. <https://doi.org/10.3897/BDJ.3.e4162>

Díaz J, Villanova GV, Brancolini F, Del Pazo F, Posner VM, Grimberg A, Arranz SE. First DNA barcode reference library for the identification of South American freshwater fish from the lower Paraná river. PLoS One. 2016.11(7), e0157419.

García‐Dávila C, Castro‐Ruiz D, Renno JF, Chota‐Macuyama W, Carvajal‐Vallejos FM, Sanchez H, Angulo C, Nolorbe C, Alvarado J, Estivals, G, Núñez-Rodríguez J, Duponchelle F. Using barcoding of larvae for investigating the breeding seasons of Pimelodid catfishes from the Marañon, Napo and Ucayali rivers in the Peruvian Amazon. J. Appl. Ichthyol. 2015. 31, 40-51.

Lima TP. Mitochondrion *Zungaro zungaro*. Submitted (DEC 2014) to the EMBL/GenBank/ DDBJ databases. 2014.

Martinez, ERM, Miguel ER, Shibatta OA, Foresti F, Oliveira C. Molecular phylogeny of Pseudopimelodidae (Teleostei: Siluriformes). Submitted (SEP-2007) to the EMBL/GenBank/ DDBJ databases. 2007.

Pereira LH, Hanner R, Foresti F, Oliveira C. Can DNA barcoding accurately discriminate megadiverse Neotropical freshwater fish fauna?. BMC Genet. 2013. 14, 1-14.

Premdass K, Lekeshmanaswamy M, Mohanchander P, Prasanna-Kumar C. DNA barcoding the freshwater ornamental fishes. Submitted (APR 2014) to the EMBL/GenBank/ DDBJ databases. 2014.

Resende LC, Carmo AO, Martins APV, Costa MA, Chagas ATA, Luz RK, Kalapothakis E. Development of genetic tools for studies with *Lophiosilurus alexandri* (Steindachner, 1876). Submitted (NOV-2015) to the EMBL/GenBank/ DDBJ databases. 2015.

Restrepo-Gómez AM, Rangel-Medrano JD, Márquez EJ, Ortega-Lara A. Two new species of *Pseudopimelodus* Bleeker, 1858 (Siluriformes: Pseudopimelodidae) from the Magdalena Basin, Colombia. PeerJ. 2020. 8, e9723.

Souza-Shibatta L, Tonini JF, Abrahao VP, Jarduli LR, Oliveira C, Malabarba LR, Sofia SH, Shibatta OA. Reappraisal of the systematics of *Microglanis cottoides* (Siluriformes, Pseudopimelodidae), a catfish from southern Brazil. PloSone. 2018.13(7), e0199963.
